# Pain modulation effect of breathing-controlled electrical stimulation (BreEStim) is not likely to be mediated by deep and fast voluntary breathing

**DOI:** 10.1038/srep14228

**Published:** 2015-09-18

**Authors:** Huijing Hu, Shengai Li, Sheng Li

**Affiliations:** 1 Department of Physical Medicine and Rehabilitation, University of Texas Health Science Center at Houston, Houston, TX, USA; 2 Neurorehabilitation Research Laboratory, TIRR Memorial Hermann Research Center, Houston, TX, USA; 3 Guangdong Provincial Work Injury Rehabilitation Center, Guangzhou, China

## Abstract

Voluntary breathing-controlled electrical stimulation (BreEStim), a novel non-invasive and non-pharmacological treatment protocol for neuropathic pain management, was reported to selectively reduce the affective component of pain possibly by increasing pain threshold. The underlying mechanisms involved in the analgesic effect of BreEStim were considered to result from combination of multiple internal pain coping mechanisms triggered during BreEStim. Findings from our recent studies have excluded possible roles of acupuncture and aversiveness and habituation of painful electrical stimulation in mediating the analgesia effect of BreEStim. To further investigate the possible role of voluntary breathing during BreEStim, the effectiveness of fast and deep voluntary breathing-only and BreEStim on experimentally induced pain was compared in healthy human subjects. Results showed no change in electrical pain threshold after Breathing-only, but a significant increase in electrical pain threshold after BreEStim. There was no statistically significant change in other thresholds after Breathing-only and BreEStim. The findings suggest that the analgesic effect of BreEStim is not likely attributed to fast and deep voluntary breathing. Possible mechanisms are discussed.

Pain, including neuropathic pain, is a subjective feeling in nature. It is multi-dimensional, including sensory, affective (i.e., unpleasantness), and cognitive components. These components are processed in parallel and inseparable to each other^1^,^2^. For example, when superficial tactile stimulation is applied to the hand area where acupuncture points are located, activation is seen only in somatosensory cortices, i.e., only tactile sensation. However, when the acupuncture points are stimulated, and pain is experienced by the subjects, activation of additional pain-related cortical areas, such as anterior cingulate cortex (ACC) and insula, is observed^3^. This is important in that sensory and affective components are processed in parallel, and therefore, these components could theoretically be modulated separately for therapeutic purposes. Neuropathic pain is difficult to manage and is associated with poorer physical, psychological and social functioning^4^,^5^,^6^. A number of non-pharmacological neurostimulation modalities have been used for management of phantom pain with various degrees of success, including transcutaneous electrical nerve stimulation (TENS)^7^, spinal cord stimulation^8^, deep brain stimulation^9^ and transcranial direct current stimulation^10^. It has also been found that repetitive painful stimulation (aversiveness) leads to significant pain attenuation^11^.

Recently, we reported a novel non-invasive and non-pharmacological treatment protocol—Breathing-controlled electrical stimulation (BreEStim) for neuropathic pain management after traumatic amputation and spinal cord injury^12^,^13^, based on our pioneering discovery that human voluntary breathing could have systemic effects on the non-respiratory systems^14^,^15^,^16^,^17^,^18^. During BreEStim, painful yet tolerable electrical stimulation is triggered and delivered transcutaneously to a peripheral nerve when a fast and deep inhalation reaches the preset inspiration threshold. In our recent case report^13^, shooting phantom pain in a patient who had an above-the-knee amputation disappeared after one week of treatment with BreEStim to the ipsilateral forearm. In this case, BreEStim to the forearm was not likely to modify the source of noxious stimuli located at the residual limb. Rather, BreEStim modified the affective response to the same stimuli such that the patient could tolerate them better, possibly by increasing pain threshold. The analgesic effect of BreEStim was considered to result from combination of multiple internal pain coping mechanisms triggered during BreEStim, possibly including habituation to aversive electrical stimulation, acupuncture effect, influence of voluntary breathing and anterograde amnesia to aversive stimulation^13^.

To investigate possible underlying mechanisms, we recently compared electrical pain thresholds before and after BreEStim and conventional electrical stimulation (EStim)^19^. Two interventions were given to the median nerve of the dominant hand of the same healthy subjects with at least 3 days apart. The intensity of painful electrical stimulation was comparable between two interventions. However, electrical pain threshold was significantly increased in both dominant and non-dominant hand after BreEStim, but decreased bilaterally after EStim. Other thresholds (thermal, electrical sensation, and tactile sensation) remained the same after both interventions. In this study^19^, electrical stimulation to the median nerve may also stimulate the acupuncture point (Neiguan) as in the original protocol^13^. Electroacupuncture had been reported to relieve pain by using electrical stimulation through needles in specific acupuncture points^20^,^21^,^22^,^23^. To further examine possible contributions of electroacupuncture to pain reduction during BreEStim, we used the same protocol^19^, but stimulated a different peripheral nerve – ulnar nerve at the elbow on the dominant side where no acupuncture points are involved. The same pattern of results was observed that electrical pain thresholds decreased after EStim, but increased after BreEStim bilaterally^24^. Collectively, these findings confirmed selective increase of electrical pain thresholds, thus pain tolerance after BreEStim. However, habituation to aversive electrical stimulation and acupuncture effect were not likely to mediate the BreEStim effect.

There are reports that voluntarily regulated breathing alone reduces pain perception^25^,^26^,^27^. For example, pain intensity and unpleasantness were reduced during slow breathing (half of normal rate) as compared to normal breathing in both healthy subjects and subjects with fibromyalgia syndrome. Though it is different from fast and deep inhalation used in BreEStim, slow breathing also requires voluntary control of breathing. It is also anecdotal observation that people usually take fast and deep breathing with acute pain, for example, after a superficial cut on a finger by a knife. However, the effect of short fast and deep breathing on pain reduction has not been studied. Its possible contribution to the analgesic effect of BreEStim remains unknown.

Therefore, the specific aim of this study was to examine the role of voluntary breathing in the analgesic effect of BreEStim using our recently established experimental paradigm in healthy human subjects^19^,^24^. We hypothesized that a short bout of fast and deep voluntary breathing could have analgesic effect, while BreEStim could have better analgesic effect than voluntary breathing alone, since it may trigger multiple internal pain coping mechanisms^13^.

## Methods

### Subjects

Eleven healthy and young adults (6 male, 5 female; age range: 28–45 years, mean: 36.5; standard deviation [SD] = 6.9) participated in the experiment. They were all right-handed according to preferred use of hand in writing and eating. None of subjects had history of neuromuscular diseases or any cardiovascular or respiratory diseases that prevented them from participating in the study. All subjects were pain free. Informed consent was obtained from all subjects prior to the experiments in compliance with a research protocol approved by the Committee for the Protection of Human Subjects at The University of Texas Health Science Center at Houston and TIRR Memorial Hermann Hospital. The methods were carried out in accordance with the approved guidelines

### Interventions

In the present study, all subjects were required to receive two interventions: BreEStim and Breathing-only. Two interventions were administered on two separate days with at least 3 days apart. The order of two interventions was randomized across all subjects to minimize the order effect. Before each intervention, a training session of approximately 5 minutes was given to ensure that subjects were familiar with the breathing methods. During both intervention sessions, voluntary breathing with deep and fast inhalation was required. The key difference between BreEStim and Breathing-only was whether or not electrical stimulation was delivered. Electrical stimulations were triggered by voluntary breathing in BreEStim sessions while no electrical stimulation was triggered in Breathing-only sessions. To examine the possible effect of voluntary breathing on pain modulation during BreEStim, the amount of quantifiable voluntary breathing (120 voluntary inhalations) was the same for both BreEStim and Breathing-only. Quantitative sensory testing (QST) was performed before and 10 minutes after each intervention to assess the effect of each intervention as in the previous studies^19^,^24^.

#### Breathing-only

**Figure 1:**
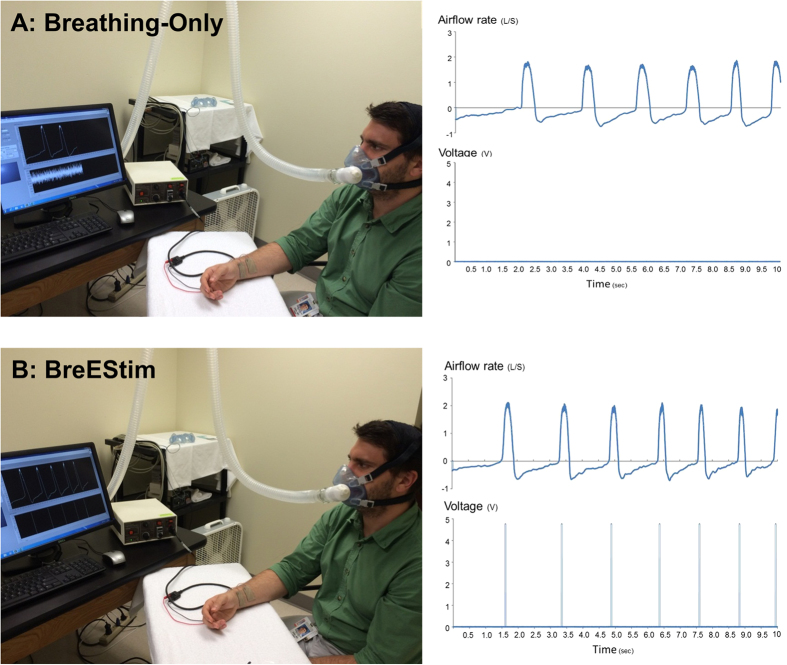
Experimental settings and representative visual feedback for Breathing-Only (A) and BreEStim (B).

In this experiment, voluntary breathing was defined as an effortful fast and deep inhalation and routine exhalation (Fig. 1). Wearing a suitable leak-prove facemask, subjects were instructed to sit comfortably with hands on an experimental table and take self-initiated fast inhalation as deep as possible. Subjects were instructed not to take effortful exhalation preceding or following the voluntary inhalation. Chest wall expansion during voluntary inhalation was often encouraged. It was similar to a routine deep breath but faster and stronger. The facemask was connected to a pneumotach system (Hans Rodulph Inc) and the airflow rate was monitored on the computer screen to ensure sufficient inhalation effort (see BreEStim session). A visual feedback of airflow rate signal and breathing patterns on the computer screen was provided for subjects to modulate the depth and rate of breathing. “Fastness” of voluntary breathing was defined by the airflow threshold of 40% of maximum airflow rate. “Deepness” of voluntary breathing was based on instruction of “as deep as possible” and chest wall expansion. Subjects may demonstrate different absolute value of airflow rate and deepness. After a practice session, we noticed similar patterns of breathing signals throughout the treatment trials between two interventions for all subjects (Breathing-only and BreEStim) (Fig. 1).

#### BreEStim

In the BreEStim session, subjects received voluntary breathing-triggered electrical stimulation. A pair of trimmed surface electrodes (2 × 2 cm^2^) was placed on the medial aspect of the distal forearm where the median nerve travels from the forearm to hand. The two electrodes were separated approximately 10 mm. An electrical stimulator (Digitimer, UK, model D185-HB4) was controlled by the experimental computer via a customized LabView (National Instrument, Austin, TX) program. Similar to the Breathing-only session, subjects were instructed to take self-initiated fast and deep inhalation without preceding or following voluntary exhalation. A single-pulse electrical stimulus would be trigged once the airflow rate reached a preset threshold (40% of its peak value) (Fig. 1) during voluntary inhalation. Details of BreEStimi intervention are available on the open access methodology video article^12^ at http://www.jove.com/video/50077/.

The intensity of electrical stimulation delivered during BreEStim started from the electrical pain threshold of individual subjects and was gradually increased to the highest level as tolerated by subjects themselves. As at such level, subjects may feel painful, annoying or noxious, but were still able to tolerate repetitive stimulation well. As reported in our previous studies^12^,^19^,^24^,^28^, the aversiveness of electrical stimulation was important during the BreEStim intervention. Verbal encouragement was given to subjects to gradually increase the intensity of electrical stimulation as tolerable as possible. The intensities of electrical stimulation were recorded at the beginning and at the 20^th^, 40^th^, 60^th^, 80^th^, 100^th^ and 120^th^ trial of the BreEStim intervention.

When wearing a facemask, subjects usually tolerated such breathing well. No hyperventilation or hypoxia stress has been reported in this study or in our previous studies^12^,^19^,^24^,^28^. During both BreEStim and Breathing-only interventions, inter-trial break was intermittently encouraged and was allowed upon request to ensure sufficient rest.

### Assessment – Quantitative Sensory Testing (QST)

QST was performed before and 10 minutes after each intervention (BreEStim and Breathing-only), including electrical sensation threshold, electrical pain threshold and thermal thresholds. These tests were performed on the thenar eminence of both dominant (treatment) and non-dominant (non-treatment) hands to investigate the central effect of interventions. The order of QST was randomized and balanced between two hands.

#### Electrical sensation and pain thresholds

The same surface electrodes were used to test electrical sensation and pain thresholds. Subjects were instructed to sit comfortably with their palms up on an experimental table. A pair of trimmed electrodes was placed on the center of thenar eminence one next to the other with 5 mm distance. The centers of thenar eminence in both hands were marked with a pen symmetrically. The borders of each electrode were marked to ensure consistence of the same position for testing before and after the intervention. For electrical sensation threshold, the intensity of electrical stimulation started from zero and was gradually increased in steps of 0.01 mA. The electrical sensation threshold was defined as the least intensity of an electrical pulse that was first detected by subjects. Three repetitions were examined and the average was calculated as the electrical sensation threshold. After the electrical sensation threshold level was detected, the intensity of stimulation was gradually increased in steps of 0.1 mA. Then electrical pain threshold was measured as the least intensity of an electrical pulse that was first felt painful by subjects. Such a painful level was equivalent to 1 on the 0–10 visual analogue scale. Similarly, three repetitions were also made and the average was used as the electrical pain threshold.

### Thermal thresholds

Thermal thresholds including warm sensation, cold sensation, heat pain and cold pain thresholds were performed using a pain and sensory evaluation system (Medoc PATHWAY model ATS). We employed the established “limits Full Series” protocol to assess the thermal thresholds. A 30 × 30 mm ATS (advanced thermal stimulator) probe (thermode) was placed in the marked center of thenar eminence. The ATS delivers painful and non-painful stimuli at a temperature range of 0 °C to 52.5 °C with heating and cooling rate of up to 8 °C/sec. Subjects were instructed to press a responding mouse button with index finger as soon as cooling or warming sensation was first perceived in order to cease the temperature changing and stimulation. That temperature level was used as cold sensation or warm sensation threshold. These thresholds were detected for 4 times, respectively. Then subjects were instructed to endure further cooling or warming until they began to feel pain, at which they need to press the responding mouse button to stop further thermal stimulation. To keep individual consistency, subjects were instructed pain threshold to be equivalent to 1 on the 0–10 VAS and were examined for three times for heat pain and cold pain thresholds, respectively.

### Data analysis and Statistical analysis

Values of electrical and thermal thresholds were measured from both dominant (treatment) and non-dominant (non-treatment) hands before and after each intervention. Analysis of these thresholds was performed using SPSS (v.16.0). Since BreEStim and Breathing-only treatments were performed on two separate days, the baseline values prior to each treatment were compared using paired t-tests. The individual thresholds across treatment were subjected to repeated-measures analyses of variance (RM-ANOVA). To evaluate the effect of each intervention on both hands, a two-way RM-ANOVA was performed with factors of TREATMENT (2 levels, pre- vs. post-) and HAND (2 levels, treatment vs. non-treatment). The effect of each intervention was quantified using the following equation: percentage change = (post-intervention − pre-intervention)/pre-intervention × 100%. To assess the effect of intervention on thresholds between BreEStim and Breathing-only, a two-way RM-ANOVA with two factors (INTERVENTION and HAND) was performed. Post-hoc Bonferrnoi comparison tests were performed when there was a significant effect. The alpha level required for all statistical significance was set at 0.05. Data are reported as mean ± standard errors within the text and in the figures.

## Results

**Figure 2:**
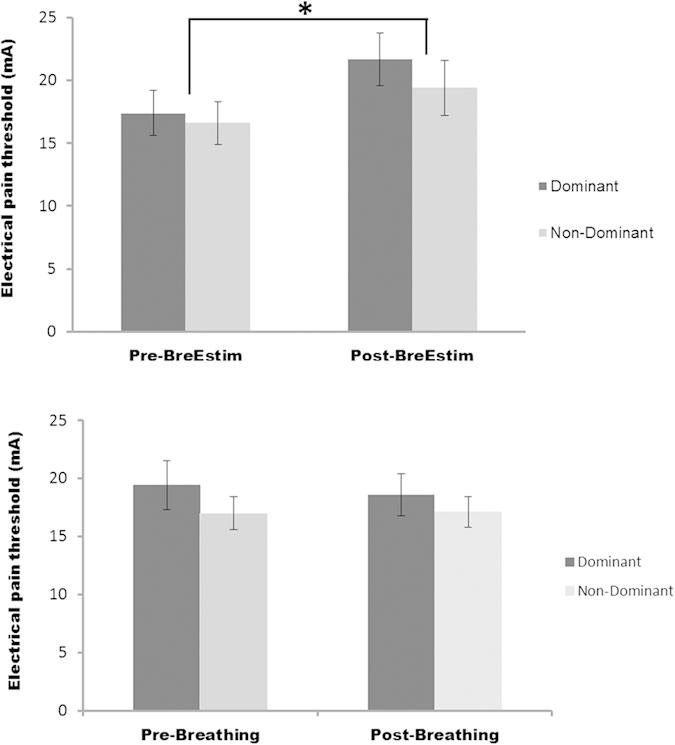
Electrical pain thresholds pre- and post-BreEstim (upper panel) and pre- and post-Breathing-only (lower panel). Asterisk indicates statistical significance. Standard errors are shown.

**Figure 3:**
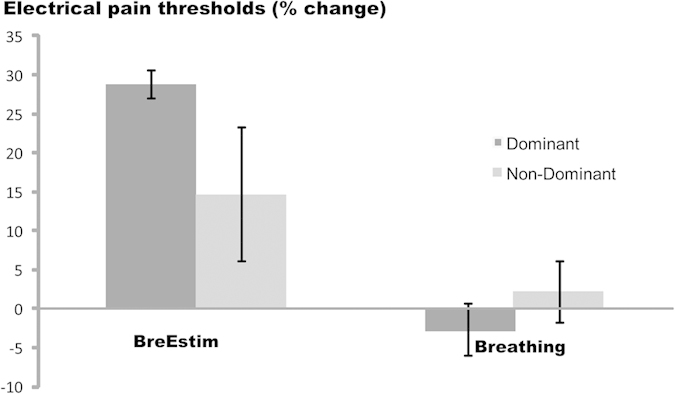
Changes of electrical pain threshold as percentage of pre-intervention values after BreEStim and Breathing-only. Standard errors are shown.

Summaries of electrical sensation threshold, electrical pain threshold and thermal thresholds are shown in Table 1. Statistical results revealed no significant differences in these thresholds between pre-BreEStim and pre-Breathing-only values (paired t-tests, p > 0.05). Stable baseline measurement indicates that any difference in these thresholds between pre- and post-intervention measurement reflects the effect of intervention. Fig. 2 shows electrical pain thresholds of both dominant and non-dominant hands as a function of treatments (pre-intervention and post-intervention) for BreEStim (upper panel) and Breathing-only (lower panel). Electrical pain threshold significantly increased after BreEStim. Two-way RM-ANOVAs revealed a main effect of TREATMENT (F_1, 10_ = 15.68, p = 0.003) and no significant main effect of HAND or TREATMENT × HAND interactions on electrical pain threshold. On average, the electrical pain threshold increased from 17.0 ± 1.2 mA pre-BreEStim to 20.4 ± 1.5 mA post-BreEStim. In contrast, electrical pain threshold was not significantly changed after Breathing-only. On average, the electrical pain threshold was 18.2 ± 1.3 mA pre-Breathing-only and 17.8 ± 1.1 mA post-Breathing-only. As shown in Fig. 3, BreEStim elicited greater increase in electrical pain threshold than Breathing-only. The BreEStim-induced electrical pain threshold increase was 28.8 ± 8.6% for the dominant hand and 14.6 ± 3.9% for the non-dominant hand, while change of electrical pain threshold was −2.7 ± 3.3% and 2.1 ± 4.1%, respectively, after Breathing-only intervention. Similar 2 × 2 INTERVENTION×HAND two-way RM-ANOVAs revealed a main effect of INTERVENTION (F_1, 10_ = 11.733, p = 0.00649), whereas main effects of HAND or INTERVENTION×HAND interaction did not reach significance. The degree of BreEStim-induced electrical pain threshold increase was different for male (26.8%) and female (14.0%) subjects, if calculated separately. However, the difference in electrical pain threshold increase was not statistically significant, according to independent t-tests (p = 0.22).

For electrical sensation and thermal thresholds, similar two-way ANOVAs were performed and no significant effects of TREATMENT or HAND was found (see Table 1 for individual values). During BreEStim, the intensity of electrical stimulation increased progressively from 20.2 mA at start to 57.9 mA at the end of 120 trials. The trend of increasing was similar to that in the previous studies^19^,^24^.

## Discussion

In the present study, we compared analgesic effects between deep and fast voluntary breathing (Breathing-Only) and voluntary breathing controlled electrical stimulation (BreEStim) to the median nerve transcutaneously on the dominant side in pain-free healthy human subjects. The results showed no significant change in electrical pain threshold after Breathing-Only and a significant increase in electrical pain threshold on both dominant and non-dominant hands after BreEStim. Electrical sensation threshold and thermal thresholds (cold sensation, warm sensation, cold pain and heat pain) remained unchanged. The amount of voluntary breathing (120 fast and deep voluntary inhalations) was the same for both interventions. The analgesic effect of BreEStim on experimentally induced pain in the present study was consistent with previous studies^19^,^24^. Selective modification of electrical pain threshold without affecting thermal pain thresholds was consistent with previous studies as well^19^,^24^,^29^. Differential modification of pain threshold was likely related to different neurophysiological pathways which were excited by electrical and thermal stimulation. Generally speaking, thermal stimulation is conveyed by small myelinated (Aδ) and smaller unmyelinated (C) fibrers^30^, while painful electrical stimulation excites large sensory fibers (Aβ) but bypasses nociceptors^31^. Slow deep breathing has been proved effective to reduce pain in the settings of laboring, nursing or in experimental pain researches^25^,^32^,^33^. The findings of no analgesic effect after fast and deep breathing (Breathing-only) were consistent with previous reports of no analgesic effects induced by normal or fast breathing^34^. These contrasting findings suggest that respiration-related analgesia and thermoregulation are likely related to phase and frequency of breathing. In particular, slow deep breathing significantly increased thermal pain tolerance with concomitantly increased parasympathetic activity. This respiration-induced analgesia and its relation to thermoregulation was not observed during rapid breathing^27^. Furthermore, in a recent study^35^, pain–related brain activity was reduced during slow breathing with fast inspiration as compared to slow breathing with slow inhalation and normal breathing with fast inspiration. However, this decreased pain related activity was dissociated from spinal nociceptive transmission, thus suggesting involvement of other supraspinal mechanisms in respiration related analgesia. Taken together, consistent observations of analgesic effects after BreEStim, but no such effect after EStim only^19^,^24^ or Breathing-only in the present study suggest that the BreEStim-induced analgesic effect is not likely attributed to fast and deep fast breathing or electrical stimulation alone. Rather, the voluntary breathing related-internal pain coping mechanisms are likely triggered by electrical stimulation during the window of voluntary breathing.

Several neuroimaging studies have shown that multiple cortical and subcortical brain areas were activated by forceful respiration, including primary motor and sensory cortex, premotor area, supplementary motor area (SMA), dorsolateral premotor cortex (DLPFC), anterior cingulate cortex (ACC), insular cortex, amygdala, basal ganglia, thalamus and cerebellum^36^,^37^,^38^,^39^,^40^,^41^,^42^. Some of these areas are activated by pain as well^1^,^43^. These shared areas were found in the same group of healthy participants who underwent conditions of dyspnea (breathlessness) and pain, including the insular cortex, ACC, amygdala and medial thalamus^44^. The insular cortex and ACC have been found to be related to affective processing of pain^45^,^46^,^47^ as well as attention and memory relevant to pain processing^48^,^49^,^50^,^51^,^52^,^53^. Memory plays an important role, particularly in chronic pain. In animal models, localized micro-stimulation to the insular cortex during peripheral aversive stimulation leads to item-specific impairment of aversive memory reconsolidation, anterograde amnesia^52^. There are case reports that patients with chronic pain reported pain relief after sudden amnesia^48^,^54^. Taken together, these studies suggest that anterograde amnesia to aversive electrical stimulation could possibly be able to account for the observed BreEStim effects. Specifically, item-specific anterograde amnesia to the stimulation occurs, when aversive stimulation is delivered during activation of the insular cortex^52^. This means that aversiveness of peripheral noxious stimulation is not remembered or decreased when the insular cortex is activated during voluntary breathing. Therefore, aversive painful electrical stimulation is felt “less unpleasant”. As such, habituation to aversive stimulation at a higher intensity is expected to be reached, subsequently increasing the analgesic effect of stimulation. Future neuroimaging studies are needed to consolidate this proposed mechanism. It is worth mentioning that we observed different degrees of BreEStim-induced electrical pain threshold increase. The gender difference was not statistically significant. This may be due to a small sample size. It could also be a reflection of variations of response among subjects. Although no definitive conclusion regarding the gender difference could be made at this time, it deserves further investigation on its own.

## Concluding remarks

In conclusion, the present study compared analgesic effects between voluntary breathing-only and BreEStim on experimentally induced pain in pain-free healthy human subjects. The results showed significantly increased pain threshold after BreEStim, but no such change after voluntary breathing-only. The findings indicate that pain modulation effect of BreEStim is not attributed to deep and fast voluntary breathing alone. Findings from our recent studies^19^,^24^ have excluded possible roles of aversiveness and habituation of painful electrical stimulation and acupuncture mediating the analgesic effect of BreEStim. Collectively, these findings suggest that the analgesic effect of BreEStim is likely due to voluntary breathing-related internal pain coping mechanisms that are triggered by electrical stimulation during the specific window of voluntary breathing.

## Additional Information

**How to cite this article**: Hu, H. *et al.* Pain modulation effect of breathing-controlled electrical stimulation (BreEStim) is not likely to be mediated by deep and fast voluntary breathing. *Sci. Rep.* **5**, 14228; doi: 10.1038/srep14228 (2015).

## Figures and Tables

**Table 1:** Quantitative measurement of thresholds of both dominant (DH) and non-dominant (NDH) hands before and after BreEStim and Breathing only. Means and standard errors (SE) are presented.

	Electrical Sensation		Electrical pain		Cold sensation		Warm sensation		Cold pain		Warm pain	
	DH	NDH	DH	NDH	DH	NDH	DH	NDH	DH	NDH	DH	NDH
preBreEstim (mean)	3.4	3.4	17.4	16.6	30.4	30.2	33.7	33.5	16.9	21.4	40.0	40.5
preBreEstim (SE)	0.3	0.2	1.8	1.7	0.2	0.3	0.2	0.2	2.1	1.8	1.0	1.1
postBreEstim (mean)	3.7	3.4	21.7	19.1	29.8	29.7	33.9	33.7	17.8	20.4	41.1	41.1
postBreEstim (SE)	0.3	0.2	2.1	2.2	0.3	0.4	0.3	0.1	2.3	1.7	1.4	1.1
preBreathing (mean)	3.5	3.5	19.4	17.0	30.6	30.2	33.7	33.9	17.0	19.8	40.8	41.4
preBreathing (SE)	0.3	0.2	2.1	1.4	0.2	0.2	0.2	0.2	2.3	2.0	0.8	0.8
postBreathing (mean)	3.5	3.5	18.6	17.1	29.9	29.7	33.7	33.8	18.7	18.5	41.5	41.6
postBreathing (SE)	0.3	0.2	1.8	1.3	0.3	0.4	0.2	0.2	1.9	2.3	0.9	1.0
